# Investigation of the Time-Dependent Deformation of Recycled Aggregate Concrete in a Water Environment

**DOI:** 10.3390/ma17184588

**Published:** 2024-09-19

**Authors:** Xingzong Liu, Bin Gong, Yufang Fu, Guanghui Jiang, Jintao Wang

**Affiliations:** 1 Key Laboratory of Transport Industry of Road Structure and Material, Research Institute of Highway, Ministry of Transport, Beijing 100088, China; liu_xingzong@163.com (X.L.); yf.fu@rioh.cn (Y.F.); 2 School of Civil Engineering, Ludong University, Yantai 264025, China; jgh@ldu.edu.cn (G.J.); wangjintao@ldu.edu.cn (J.W.); 3 College of Engineering, Design and Physical Sciences, Brunel University London, London UB8 3PH, UK

**Keywords:** time-dependent deformation, recycled aggregate concrete, long-term strength, water environment, confining pressure effect

## Abstract

The water environment greatly affects the creep deformation of recycled aggregate concrete (RAC). Hence, a humidity–stress–damage coupling numerical model was used for investigating the time-dependent deformation of RAC in the water environment in this study. Firstly, uniaxial compression and water absorption tests were performed to determine the calculation parameters of the creep numerical simulation of RAC in a water environment. Experimental results indicate that the elastic modulus and compressive strength drop as the water content increases. Then, the time-dependent deformation of RAC in a water environment was studied using a numerical simulation test of compressive creep when multiple stress levels were applied, and the critical stress for accelerated creep and the long-term strength of RAC were obtained. Finally, the influence of confining pressures on the long-term deformation of RAC in a water environment was discussed. When there is no confining pressure, the long-term strength of RAC is 23.53 MPa. However, when a confining pressure of 3.921 MPa is loaded onto RAC, the long-term strength of RAC is 47.052 MPa, which increases by 100%. Increasing confining pressures has an obvious effect on ensuring the long-term stable application of RAC in a water environment. Compared with the creep test, the method adopted in this study saves time and money and provides the theoretical basis for evaluating the time-dependent deformation of RAC in a water environment.

## 1. Introduction

A large amount of waste concrete from construction and demolition waste is produced worldwide each year [1]. The use of recycled aggregate (RA) is a crucial step for sustainable development in the concrete industry and the effective management of construction waste [2]. Although some countries have regulations permitting the use of RA in concrete production, even on a small scale, its actual implementation remains uncommon [3]. Numerous studies have focused on analyzing and characterizing RA, as well as evaluating the performance of concrete made with it. These studies indicate that while the use of RA reduces the mechanical performance of concrete, it has a more significant impact on its durability-related properties [4,5,6,7,8,9,10,11]. However, some research showed that RA can be acceptable in concrete production. Huang et al. (2021) [12] indicated that recycled fine aggregate (RFA) can be used as fine aggregate in alkali-activated slag concrete (AASC) with better compressive strength at an early stage in comparison to river sand. Xiao et al. (2021) [13] demonstrated that incorporating up to 30% coarse recycled concrete aggregate did not affect concrete strength, though a gradual decline was observed as the content increased beyond that point. Tam et al. (2007) [14] introduced a two-stage mixing approach to enhance the strength of RAC, making it viable for higher-grade applications. Yehia et al. (2015) [2] found that RAC with reasonable strength and durability can be achieved if a high packing density is maintained. Soares et al. (2014) [15] conducted extensive tests to assess the impact of incorporating recycled aggregates from crushed precast concrete elements. Due to the high quality of the coarse aggregates recycled from these elements, the results showed that concrete mixes with recycled aggregates performed equivalently to pure concrete in terms of most properties.

Because of the complicated composition and varying parameters of the recycled materials, it is challenging to calculate the post-construction settlement and ensure the long-term stability of engineering that adopts the recycled materials. At present, scholars pay more attention to the short-term behavior and strength parameters of concrete [16,17,18]. However, RA generally has a more significant impact on time-dependent deformation than on the short-term behavior and parameter strength of concrete. The incorporation of RA in concrete leads to a noticeable increase in creep and shrinkage, which influences the long-term performance of concrete structures. In order to ensure the safe application of RAC structures, the time-dependent deformation characteristics of RAC should be studied because this material can provide great environmental and economic benefits. To date, there is a significant amount of research to assess the creep and shrinkage of RACs. Domingo et al. (2010) [19] found that replacing 100% of coarse natural aggregate with recycled aggregate led to a 51% increase in creep deformation and a 70% increase in shrinkage in comparison to conventional concrete. Knaack and Kurama (2015) [20] conducted an experimental study on the time-dependent sustained service-load behavior of normal-strength concrete beams containing recycled concrete aggregates. Their results showed that higher amounts of RA significantly increased both immediate and long-term deflections in concrete beams, although this effect was less pronounced in beams with increased cracking. Seo and Lee (2015) [21] performed a uniaxial restrained shrinkage cracking experiment to study the tensile creep parameters resulting from the restraint of drying shrinkage in RAC. Liu et al. (2019) [22] conducted a 1200-day long-term deformation experiment and developed the related formula for computing the long-term deformation of recycled concrete. Sryh and Forth (2022) [23] pointed out that higher levels of RA led to stronger creep and shrinkage in RAC, as well as a greater long-term loss of tension stiffening in reinforced tension samples.

The creep of RAC is a key index for estimating the long-term deformation of RAC structures. At present, conducting experiments is a common approach to investigate the time-dependent deformation of RAC. Experimental testing to investigate the behavior of RAC under certain loads is both costly and time-consuming. Therefore, developing a reliable method to study the long-term behavior of RAC is highly desirable [24]. With the development of computer technology, some scholars tried to reveal the long-term deformation characteristics of RAC using numerical methods. Knaack and Kurama (2020) [25] described numerical parametric research focusing on the influence of coarse RA on the time-dependent vertical deflections of reinforced concrete columns subject to sustained service loads. Geng et al. (2016) [26] established a finite element model with ABAQUS to study the time-dependent response of steel tubular columns filled with recycled coarse aggregate concrete. Tosic and Kurama (2020) [27] developed a concrete creep and shrinkage model to carry out a service-load deflection analysis of a RAC one-way slab and T-beam. Guo et al. (2019) [28] proposed a coupled creep–damage model to study the creep of recycled aggregate concrete, considering the old attached mortar. Immersion can cause particle lubrication and softening, meaning that changes in the external environment can significantly affect the long-term deformation of structures made from recycled materials [29,30,31]. However, few studies have been performed on the time-dependent behavior of RAC in a water environment.

In this study, with the aim of characterizing the time-dependent deformation of RAC in a water environment, a humidity–stress–damage coupling numerical model was used to clarify the influence of numerous stress levels and confining pressures on the time-dependent deformation of RAC. Furthermore, the long-term strength and critical stress for accelerated creep of RAC in a water environment were determined by numerical simulation.

## 2. Materials and Methods

Realistic failure process analysis (RFPA), as a finite element-based method, is able to capture the fracturing processes of quasi-brittle materials, e.g., rock or concrete [32,33,34,35]. The fundamental assumption for RFPA is that the heterogeneity of material strength leads to its progressive failure. To model the random microcracks inside the material, material heterogeneity is characterized using the statistical approach [36,37,38,39]. In this study, the statistical distribution of material mechanical parameters is characterized using the Weibull distribution function [40,41], as detailed below:

(1) $$\varphi \left(f\right)=\frac{m}{f_{0}}{\left(\frac{f}{f_{0}}\right)}^{m-1}\exp{\left[-\left(\frac{f}{f_{0}}\right)\right]}^{m-1}$$

where *m* represents the shape of a Weibull distribution curve and is termed the homogeneity index, *f* denotes the mechanical parameters, including the uniaxial compressive strength and elastic modulus, and *f*_0_ represents the mean value of the mechanical parameters of elements. Based on the Weibull distribution, the homogeneity index *m* determines the shape of the density curve and implies the homogeneity degree of the material. A larger *m* value means that more elements have mechanical property values that approximate the mean value, indicating a more homogeneous material sample.

RFPA2D-Humidity is the two-dimensional humidity diffusion version of RFPA. In RFPA2D-Humidity, the finite element method (FEM) is used for the stress and strain analyses. Meanwhile, a humidity–stress/strain–damage (HSD) model is adopted to represent the relationship between stress/strain, humidity, and damage. The principles of the HSD model are as follows:

(1) The mechanical properties (elastic modulus, Poisson’s ratio, and strength) of all elements are assumed to obey the given Weibull distribution to express the heterogeneity of the material.
(2) The material is elastic-brittle, and the mechanical behavior of the material follows the elastic damage constitutive rule.
(3) One element fails in tensile mode if the minimum principal stress is lower than the negative tensile strength, whereas an element fails in shear mode if the shear stress meets the Mohr–Coulomb strength criterion.
(4) The main concern is the change in the mechanical phenomena of the material over the wetting time, and the complex chemical actions during the moisture diffusion of the material are neglected.
(5) Dilation and softening induced by moisture diffusion in the material are similar to those induced by the thermal effects, although the physical mechanisms are different.

In RFPA2D-Humidity, the humidity transport within the material changes the humidity field. Simultaneously, dilation and softening of the material occur due to the change in the humidity distribution. Dilation and softening influence the stress and deformation fields.

For unsaturated materials, the moisture concentration gradient, i.e., Fickian transport, is effective for quantifying the movement of moisture [42]. Bazant and Najjar (1971) [43] applied Fick’s diffusion laws to relative humidity and represented the moisture potential using the Kelvin–Laplace equation. Moisture diffusion in the material under the moisture gradients is defined by Equation (2):

(2) $$\frac{\partial h}{\partial t}=\nabla \left(D_{h}\nabla h\right)$$

where ∇ represents the gradient or del-operator; *h* denotes relative humidity; *t* is time, and *D_h_* represents the humidity diffusion coefficient, which cauterizes the humidity degree, as well as damage evolution. As we all know, the growth in humidity indicates an increase in moisture content, and the saturation of the material also increases qualitatively. In this study, humidity is likened to saturation in the material, and Equation (2) can be rewritten as

(3) $$\frac{\partial S_{r}}{\partial t}=\nabla \left(D_{h}\nabla S_{r}\right)$$

where *S_r_* is saturation. The softening of the material is modeled as follows:

(4) $$X=\{\begin{array}{c} X_{0}\;\;\;S_{r}<S_{\mathrm{rc}}\\ X_{0}(1-\frac{S_{rt}-S_{rc}}{1-S_{rc}}\xi )\;\;\;S_{r}\geq S_{\mathrm{rc}} \end{array}$$

where *X*_0_ and *X* are the initial and current mechanical parameters (strength, elastic modulus, and Poisson’s ratio), respectively. *S_rt_* represents the current saturation. *S_rc_* is a critical saturation value, which means that because the saturation is larger than *S_rc_*, the degradation of material parameters caused by the humidity diffusion should be taken into account. *ξ* represents the degradation coefficient for mechanical parameters with saturation.

Miao et al. indicated that the dilation of rock-like quasi-brittle material under humid conditions can be simulated similarly to that of the thermal expansion [44]. The strain caused by humidity diffusion can be written as

(5) $$\varepsilon_{h}=\beta \left(S_{r}\right)\Delta S_{r}$$

where *ε_h_* represents humidity strain, meaning the strain induced by the change in humidity in the material, and *β(S_r_)* represents the swelling coefficient. Δ*S_r_* is the change in saturation. The total strain due to the coupling of stress and the humidity field includes elastic strain and humidity strain. The constitutive and equilibrium equations of the material, considering the coupling of external loading and humidity stress, can be expressed by

(6) $$\varepsilon_{ij}=\varepsilon_{ij}^{\sigma }+\varepsilon_{ij}^{h}$$

where *i*, *j* = 1, 2; *ε_ij_* represents the strain component, $\varepsilon_{ij}^{\sigma }$ is the elastic strain component, and $\varepsilon_{ij}^{h}$ is the humidity strain component.

According to the elastic damage constitutive law, the elastic modulus of an element gradually decreases as damage progresses, with the elastic modulus of the damaged element defined as follows:

(7) $$E=\left(1-D\right)E_{0}$$

where *D* is termed the damage variable and ranges from 0 for undamaged material to 1 for completely damaged material. *E* and *E*_0_ represent the elastic moduli of the damaged and undamaged elements, respectively.

**Figure 1:**
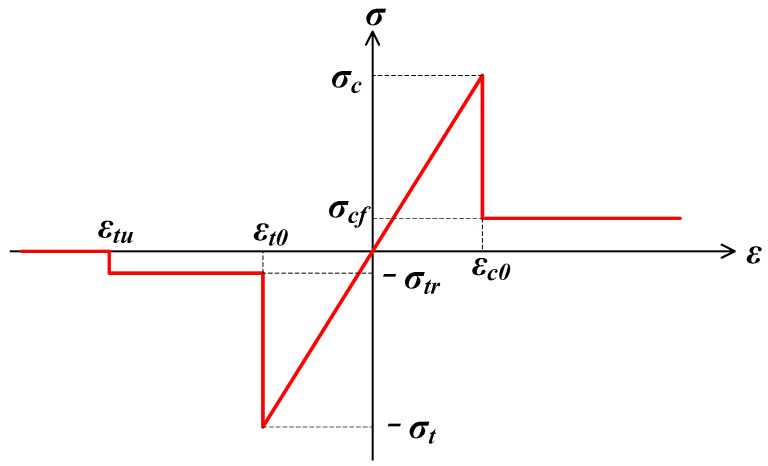
Elastic damage constitutive law.

Figure 1 shows the elastic-brittle damage constitution with a specific residual strength. If the tensile stress of one element is lower than its negative tensile strength *σ_t_*, expressed as

(8) $$\sigma_{3}\leq -\sigma_{t}$$

where *σ*_3_ represents the minimum principal stress, then the damage variable *D* can be calculated by

(9) $$D=\{\begin{array}{cc} 0 & \varepsilon <\varepsilon_{t0}\\ 1-\frac{\sigma_{tr}}{E_{0}\varepsilon } & \varepsilon_{t0}\leq \varepsilon <\varepsilon_{tu}\\ 1 & \varepsilon \geq \varepsilon_{tu} \end{array}$$

where *σ_tr_* represents the residual strength, *ε_t_*_0_ represents the tensile strain when the failure occurs, and *ε_tu_* represents the ultimate tensile strain. When elements are subject to compressive or shear stress states, the Mohr–Coulomb criterion will be applied as the failure criterion:

(10) $$\sigma_{1}-\sigma_{3}\frac{1+sin\varphi }{1-sin\varphi }\geq \sigma_{c}$$

where *σ*_1_ represents the maximum principal stress; *φ* represents the friction angle; and *σ_c_* represents the compressive strength. Then, the damage variable can be calculated by Equation (11):

(11) $$D=\{\begin{array}{cc} 0 & \varepsilon <\varepsilon_{c0}\\ 1-\frac{\sigma_{cr}}{E_{0}\varepsilon } & \varepsilon \geq \varepsilon_{c0} \end{array}$$

where *σ_cr_* represents the residual compressive strength and *ε_c_*_0_ represents the maximum principal strain.

Tang et al. (2018) [45] conducted an experiment to validate the HSD model of RFPA2D-Humidity and satisfactory agreement was obtained between the experimental and numerical results. Some scholars used the HSD model to study the time-dependent deformation of tunnels surrounding rock mass and suffering humid conditions [45,46]. These research results provide technical support for this study.

## 3. Determination of the Material Parameters

### 3.1. Homogeneity Index

**Figure 2:**
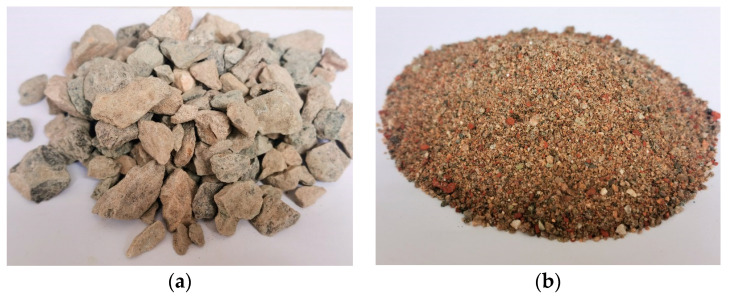
Images of aggregate. (**a**) Natural coarse aggregate. (**b**) Recycled fine aggregate.

Grade 52.5 Ordinary Portland cement was chosen here. Crushed dolomites with the maximum particle size of 25 mm were utilized as the coarse aggregate (Figure 2a). The recycled fine aggregate in this test primarily originated from urban demolition. First, the construction solid waste was screened to remove the impurities. The construction solid waste was then crushed to obtain fine granular materials. Finally, the fine granular materials were washed to remove impurities and the recycled fine aggregate was obtained. Moreover, the particle size of the recycled fine aggregate ranges from 0.35 mm to 0.5 mm (Figure 2b).

**Figure 3:**
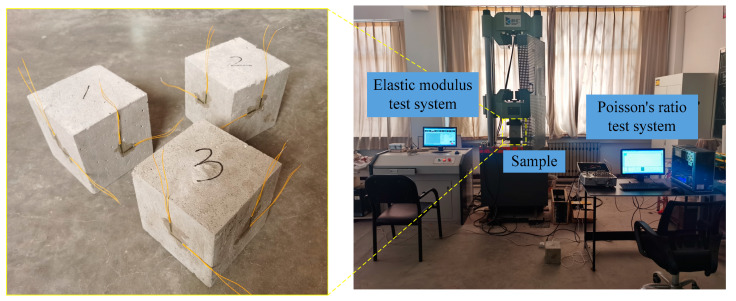
A view of the sample for the UCT.

The content of the mixed design components for pouring 1 m^3^ recycled aggregate concrete is displayed in Table 1. According to the recommendations of the Chinese code for the design of concrete structures (No. GB 50010-2010) [47], the specimen size of 150 mm × 150 mm × 150 mm was selected for the uniaxial compression test (UCT). Specimens were cured under standard conditions and were referred to as the cured specimens. Strain rosettes were glued on the lateral side of specimens, as illustrated in Figure 3.

As a result, the stress–axial strain and axial strain–lateral strain curves are obtained for every specimen. From the stress–axial strain curve, the elastic modulus of the experimental RAC can be calculated as the slope of the stress–axial strain curve. Meanwhile, Poisson’s ratio is the ratio of lateral strain to axial strain, and the compressive strength is the peak of the stress–strain curve. UCTs were performed using the displacement-controlling approach with a loading rate of 0.1 mm/min using the WAW-1000B universal testing machine produced by the Jinan Kehui Test Equipment Co., Ltd. in Jinan, China. As shown in Table 2, the elastic modulus of the experimental RAC in this research was 12,311.4 MPa on average, while the compressive strength was 39.21 MPa on average. Poisson’s ratio reached up to 0.27, averaging 0.25.

The three-dimensional version of RFPA was applied to establish a 3D numerical calculation model with a size of 150 mm × 150 mm × 150 mm. The model was divided into 421,875 hexahedral finite elements. The displacement-controlling load was applied on the top of the model, and the bottom boundary of the numerical calculation model was fixed in the normal direction. To determine the homogeneity index of the experimental RAC, a series of numerical calculations were performed with homogeneity index *m* varying from 1 to 8. The properties of numerical models were identical, and the physical and mechanical parameter values used in the calculation were obtained by UCTs. The calculated data are summarized in Table 3. When the homogeneity index is 7, the calculated elastic modulus and compressive strength are closest to the testing value. Therefore, it can be considered that the homogeneity index of the experimental RAC is 7.

### 3.2. Water Content

A water absorption experiment was conducted to investigate the change law of water content with the time of RAC specimens. Firstly, the mass of the cured RAC specimens was weighted. Then the cured RAC samples were dried in an oven at 105 °C for 48 h to a constant mass and referred to as dried specimens. After the dried specimens cooled to room temperature, the mass of the dried specimens was weighed. Moreover, the water content (*ω*) of the cured RAC specimens is the ratio of the water mass of the cured sample to the mass of the dried sample, expressed as

(12) $$\omega =\frac{m_{0}-m_{d}}{m_{d}}\times 100\%$$

where *m*_0_ is the mass of the cured specimen and *m_d_* is the mass of the dried specimen. After the test, the water content of the cured specimen is 2.75% on average. The drying process may cause the shrinkage of the RAC and affect water absorption and specimen strength. The drying method should be avoided to obtain the mass of the dried sample. Therefore, the mass of the dried specimen can be calculated according to the mass and water content of the cured specimen. Three cured specimens were immersed in water to record the mass of the samples at different immersion times. The water content is the ratio of the water mass of the specimen to the mass of the dried specimen, expressed as

(13) $$\omega_{t}=\frac{m_{w}-m_{d}}{m_{d}}\times 100\%$$

where *ω_t_* is the water content of specimens after a certain time of water absorption and *m_w_* is the mass of the sample after absorbing water.

**Figure 4:**
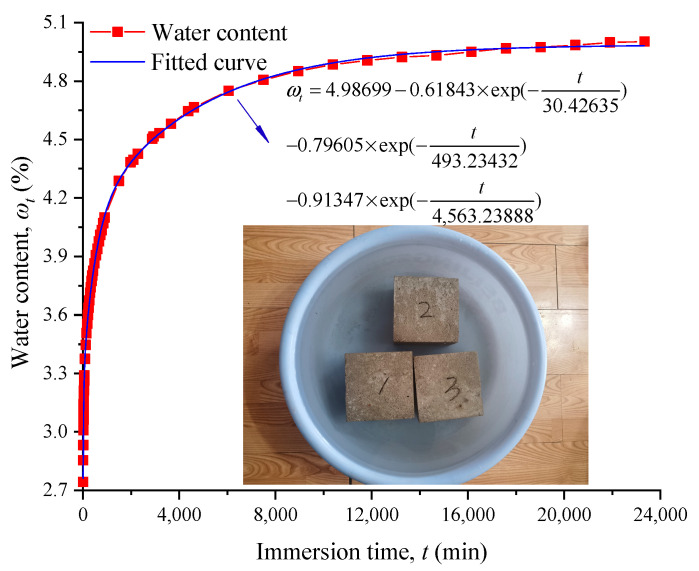
Water absorption curve.

Figure 4 indicates the correlation between the average water content of the three samples and the immersion time. The water absorption process can be divided into three stages, i.e., the quick absorption stage, the slow absorption stage, and the stable absorption stage. Moreover, the water content rapidly increased in the first stage for approximately 15 h. Then, the rate of water absorption gradually decreased and eventually remained constant after immersion in water for 365 h. The average water content of the saturated sample was 4.88%.

According to Figure 4, the relation equation between water content and time is as follows:

(14) $$\omega_{t}=4.98699-0.61843\times \exp\left(-\frac{t}{30.42635}\right)-0.79605\times \exp\left(-\frac{t}{493.23432}\right)\;-0.91347\times \exp\left(-\frac{t}{4,563.23888}\right)$$

where *t* is the immersion time.

### 3.3. Degradation Coefficient for Mechanical Properties

**Figure 5:**
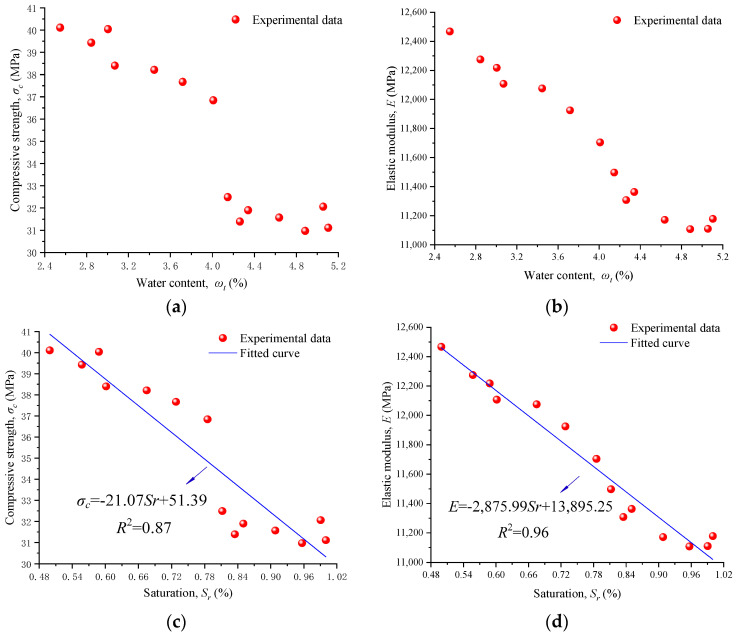
Influence of the water content on the compressive strength and elastic modulus of RACs. (**a**) Compressive strength and water content. (**b**) Elastic modulus and water content. (**c**) Compressive strength and saturation. (**d**) Elastic modulus and saturation.

According to Equation (14), 14 cured samples were immersed in water for different lengths of time to obtain specimens with different water contents ranging from 2.5% to 5.1%. Then, UCTs subjected to the same loading conditions were carried out to study the change law of elastic modulus and compressive strength with water content. The effect of water content on the compressive strength and elastic modulus of RACs is shown in Figure 5a,b. The elastic modulus and compressive strength decrease with the increases in water content. It is worth noting that when the water content exceeds 4.0%, the elastic modulus and compressive strength decrease significantly. Considering that the water content of the saturated sample is 4.88%, the critical saturation of the experimental RAC is 82%.

Figure 5c illustrates the correlation between saturation of the specimens and compressive strength. Compressive strength and saturation can be fitted by a linear equation:

(15) $$\sigma_{c}=-21.07S_{r}+51.39$$

where *σ_c_* and *S_r_* are the compressive strength and saturation of the specimen, respectively. Similarly, according to Figure 5d, the fitting equation of the elastic modulus and the saturation can be written as:

(16) $$E=-2875.99S_{r}+13,895.25$$

where *E* is the elastic modulus of the specimen. Combined with Equations (4), (15), and (16), the degradation coefficients of elastic modulus and compressive strength can be obtained as 0.04 and 0.09, respectively.

### 3.4. Humidity Diffusion Coefficient

**Figure 6:**
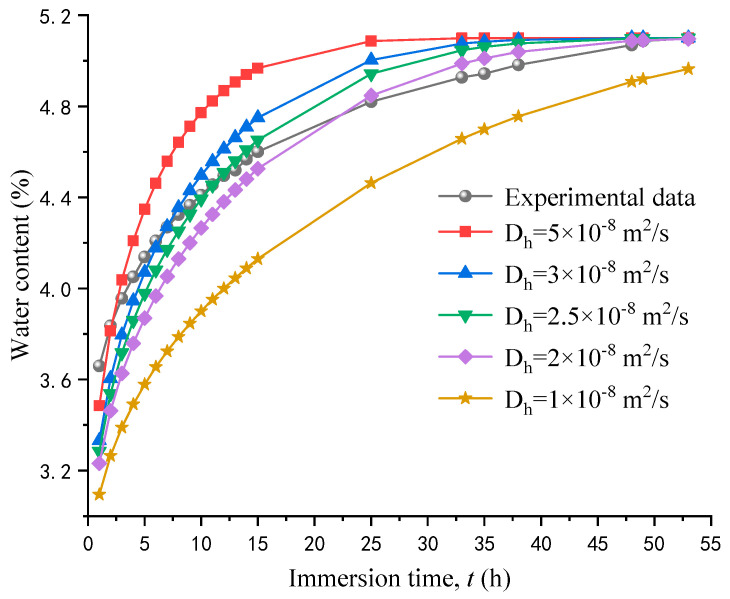
Comparison between the water absorption experimental results and the numerical simulation results under different humidity diffusion coefficients.

Water absorption tests were simulated by RFPA2D-Humidity to obtain the humidity diffusion coefficient of experimental RACs. The size of the numerical calculation model was 150 mm × 150 mm. The specimen was discretized to form 22,500 four-node quadrilateral simplex elements. Simultaneously, the boundary of the model was in contact with water, and the ambient humidity was 100%. Other parameters for calculation are listed in Table 4. To determine the humidity diffusion coefficient of the experimental RAC, a series of numerical calculations were performed with different humidity diffusion coefficients (*D_h_*). The comparison between the water absorption test results and the numerical simulation results under different humidity diffusion coefficients is shown in Figure 6. This paper mainly studies the long-term variation rule of RAC characteristics in a water environment. When the humidity diffusion coefficient is 2 × 10^−8^ m^2^/s, the numerical simulation data of the long-term water content basically coincide with the test results. Therefore, the humidity diffusion coefficient of the experimental RAC in the numerical simulation is selected as 2 × 10^−8^ m^2^/s in this paper.

## 4. Time-Dependent Deformation in a Water Environment

### 4.1. Test Process

**Figure 7:**
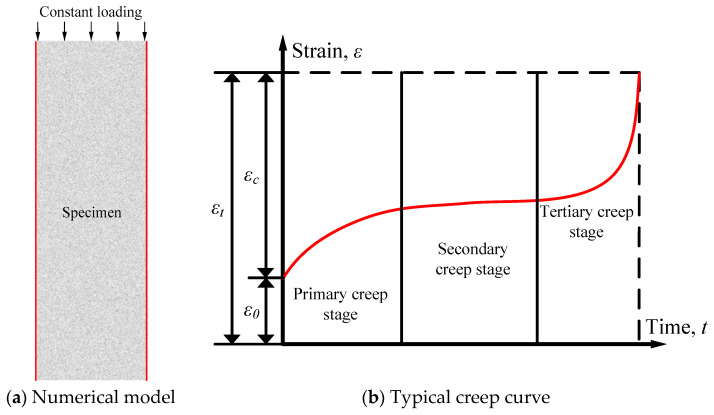
Numerical model of creep in water and the typical creep curve of the material, with three creep stages.

The time-dependent deformation of RAC in a water environment mainly occurs because of the corrosion caused by humidity diffusion. In this section, RFPA2D-Humidity was used to study the time-dependent deformation of RAC in a water environment. The numerical model was established according to the recommendations of the Chinese test code for hydraulic concrete (No. SL/T 352-2020), as illustrated by Figure 7a. The size of the model was 150 mm × 450 mm. Simultaneously, the model was discretized into 67,500 four-node elements. Constant uniaxial compression stress was arranged at the top of the model, while the bottom of the model was restrained in the vertical direction. The left and right boundaries of the model in contact with water are indicated by the red lines in Figure 7a. According to the test conditions, the top and bottom boundaries of the model were sealed, and the left and right boundaries were subjected to high saturation conditions with *S_r_* = 100%. The mechanical and diffusion parameters applied for simulation are listed in Table 4. To investigate the influence of different stress levels on the time-dependent deformation of RAC in water, eight stress levels were applied to the model. The stress levels are 5%, 10%, 20%, 30%, 40%, 50%, 60%, and 70% of the compressive strength of RAC. In addition, Figure 7b shows a typical creep curve under a single constant loading. There are three distinct stages (primary creep, secondary creep, and tertiary creep) and three strains (initial strain *ε*_0_, creep strain *ε_c_*, and total strain *ε_t_*) in the typical creep curve. The initial strain is influenced by the stress level, and the total strain equals the sum of the initial strain and creep strain. The total strain increases at a decelerating rate at the primary creep stage, while the total strain has a linear relationship with time at the secondary creep stage. The total strain increases exponentially at the tertiary creep stage, resulting in the failure of the specimen.

### 4.2. The Evolution of Strain

**Figure 8:**
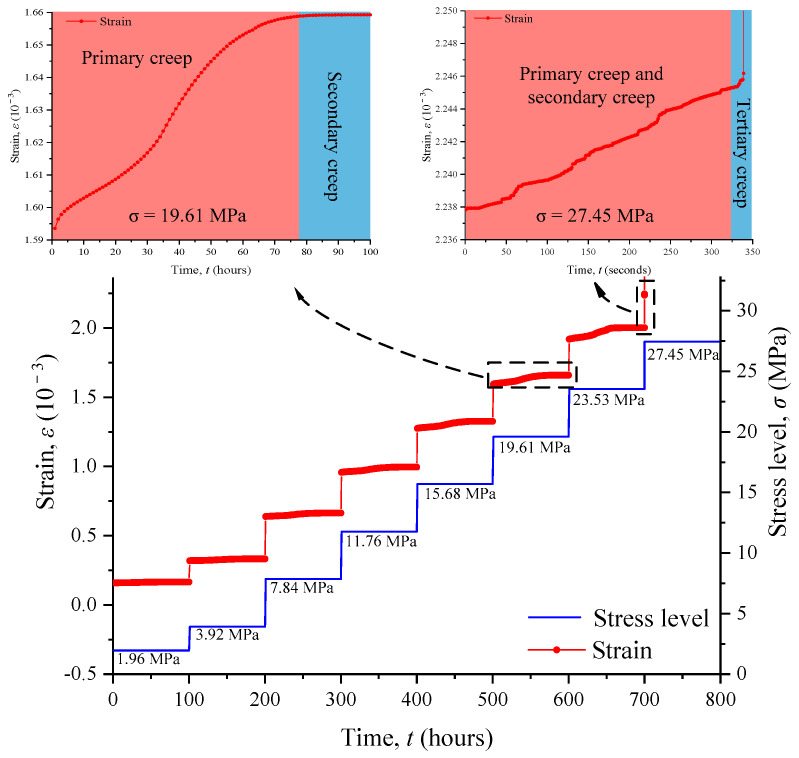
Numerical simulation results of creep tests.

The strain–time curves under different stress levels are summarized in Figure 8. If the stress level is less than 60% of the compressive strength, only the primary and secondary creep stages exist, and the tertiary creep stage does not appear. An enlarged strain–time curve when the stress level is 19.61 MPa is shown in Figure 8. The strain at the primary creep stage does not rise at a decelerating rate as the typical creep curve does. At the primary creep stage, the strain first increases with a decreasing rate, then increases with a steady rate, and finally increases with a decreasing rate. The strain–time curve is in the shape of a saddle, and the saddle-shaped trend becomes more obvious with the increase in stress level. The saddle-shaped strain–time curve was revealed by Xu et al. (2021) [48] and Li et al. (2021) [31] as well. A credible interpretation of the saddled-shaped strain–time curve may be obtained from the perspective of pore compression. At the initial period of loading, the pores are compressed and the strain increases rapidly. This process lasts for about 5 h when the stress level is 19.61 MPa. However, as the pores are compacted, strain growth is difficult and the increasing rate drops. Subsequently, the microstructure of RAC changes and creep deformation occurs, and the strain increases with a steady rate and a decreasing rate. The strain stabilizes at a seemingly constant value in the secondary creep stage. In addition, Figure 8 also indicates the strain–time relationship when the stress level is 27.45 MPa. Under this stress level, the strain increases with time and the secondary creep stage is not obvious. When the loading time exceeds 325 s, the strain increases abruptly, and the model fails.

### 4.3. Long-Term Strength

Evaluating the long-term strength of RAC in a water environment is very important to ensure the long-term stability of projects using RACs. Similar to rock, RACs may also be damaged due to creep under long-term loading. That is to say, the strength of RACs will gradually decrease with the extension of loading time. Research indicates that there is a threshold strength below which the RACs will cease to deform [49]. This threshold strength is defined as the long-term strength of RACs. According to the above analysis, stable creep will happen when the stress level is less than 23.53 MPa, and unstable creep appears when the stress level is larger than 27.45 MPa. Hence, the long-term strength (*σ_∞_*) satisfies the following formula:

(17) $$23.53\;\mathrm{MPa}\;\leq \sigma_{\infty }<27.45\;\mathrm{MPa}$$

In this paper, the long-term strength of the RAC in water is regarded as 23.53 MPa, which is about 60% of the uniaxial compressive strength of 39.21 MPa. More accurate long-term strength values can be obtained by selecting more stress level values between 23.53 MPa and 27.45 MPa for numerical creep simulation. The lower value can be treated as the long-term strength value for safety reasons in engineering practice. In practical engineering, when the stress level is over the long-term strength value, the recycled concrete may appear to be at the tertiary creep stage, leading to the failure of the samples. This phenomenon should be paid attention to.

### 4.4. Critical Stress for Accelerated Creep

It is generally accepted that there is a critical stress that corresponds to the onset of accelerated creep. Meanwhile, the determination of this critical stress is essential for the practical project. Initial strain *ε*_0_, creep strain *ε_c_*, and total strain *ε_t_* under different stress levels are listed in Table 5. According to Table 5, the creep strain proportional coefficient can be determined as the ratio of the creep strain to the total strain, expressed as Equation (18):

(18) $$\lambda =\frac{\varepsilon_{c}}{\varepsilon_{t}}$$

where λ is the creep strain proportional coefficient.

**Figure 9:**
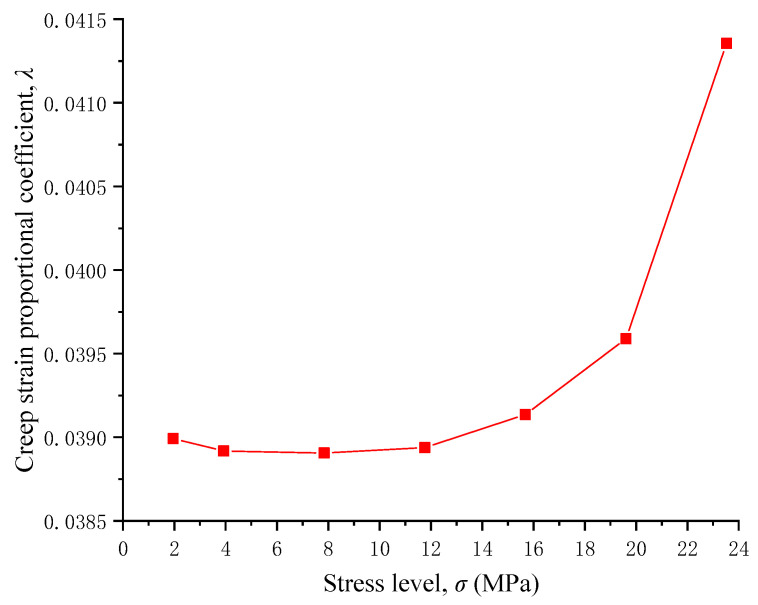
Creep strain proportional coefficient under different stress levels.

The creep strain proportional coefficient reflects creep characteristics, the decrease in the creep strain proportional coefficient indicates deceleration creep, and the increase in the creep strain proportional coefficient reflects accelerated creep [50]. Figure 9 indicates the creep strain proportional coefficient under various stress levels. With the increase in stress level, the creep strain proportional coefficient first decreases and then increases. The minimum value of the creep strain proportional coefficient appears when the stress level is 7.842 MPa. The critical stress for accelerated creep is 7.842 MPa, which is approximately 20% of the uniaxial compressive strength of RAC. If the stress level is larger than the critical stress, the proportion of creep strain in the total strain increases, and the creep deformation becomes more and more obvious. Therefore, if the stress level is larger than the critical value, attention should be paid to deformation monitoring in practical projects.

### 4.5. The Evolution of Strain under Confining Pressures

**Figure 10:**
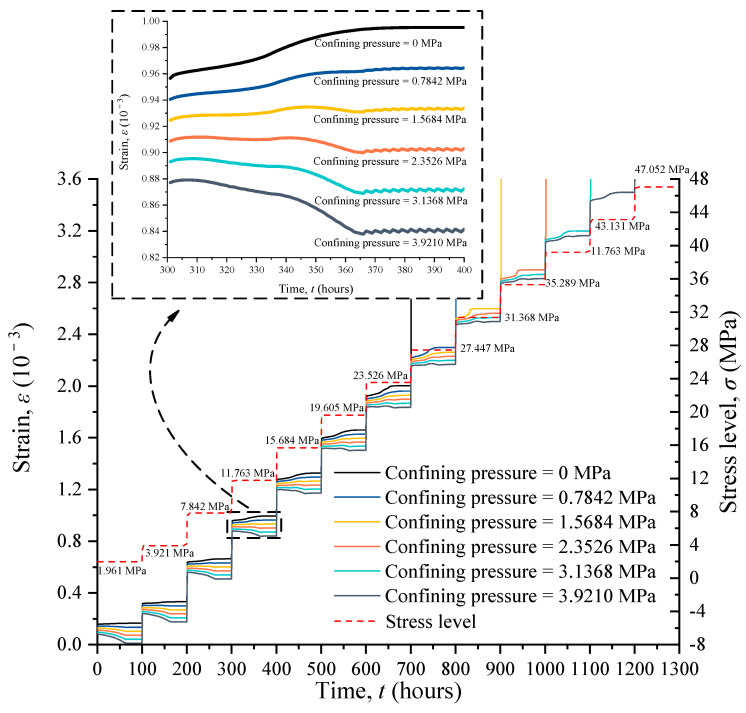
The evolution of strain under different confining pressures.

In order to investigate the effects of confining pressures on the time-dependent deformation of RAC in a water environment, a group of creep numerical simulations were conducted using several different confining pressures. The strain–time curves with various confining pressures are summarized in Figure 10. When suffering confining pressure, the horizontal strain of the sample increases and the vertical strain decreases. When the vertical constant load is small, the vertical strain does not increase in the shape of a saddle but decreases first and then becomes stable. When the vertical constant load is large, the vertical strain still increases in a saddle shape. The creep deformation of RAC is influenced by both vertical constant load and confining pressures. When the vertical constant load is applied on RAC, the confining pressure can be used to reduce the creep deformation.

**Figure 11:**
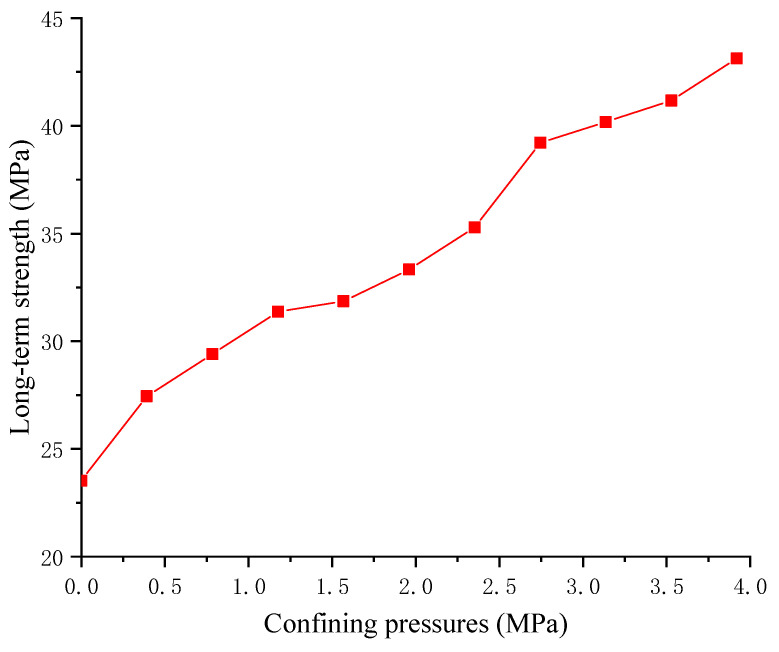
The relationship between confining pressures and long-term strength.

**Figure 12:**
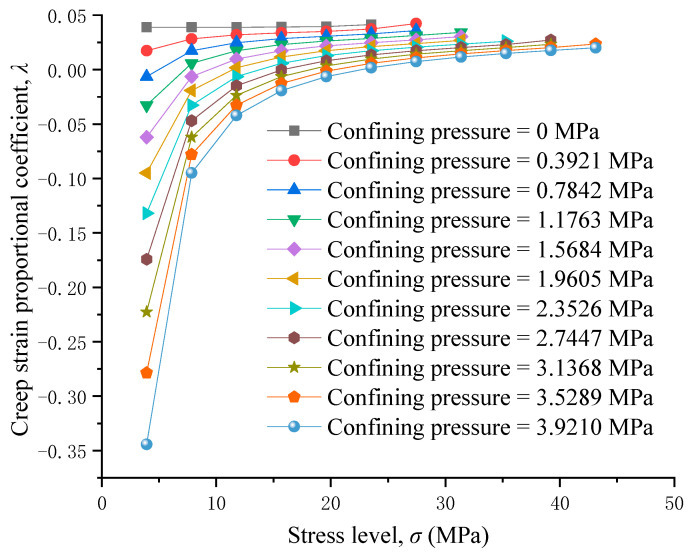
Creep strain proportional coefficient under different confining pressures.

Figure 11 reflects the correlation between confining pressure and the long-term strength of RAC. When increasing the confining pressure, the long-term strength of RAC obviously increases. The long-term strength of RAC is 23.53 MPa when there is no confining pressure, and when the confining pressure of 3.921 MPa is loaded onto RAC, the long-term strength of RAC is 47.052 MPa, which increases by 100%. Confining pressures can effectively improve the long-term strength of RAC. In practical engineering, the method of increasing confining pressures can be utilized to increase the long-term strength of RAC according to the requirements, so as to guarantee its long-term stability. The effect of confining pressures on the creep strain proportional coefficient is shown in Figure 12.

**Figure 13:**
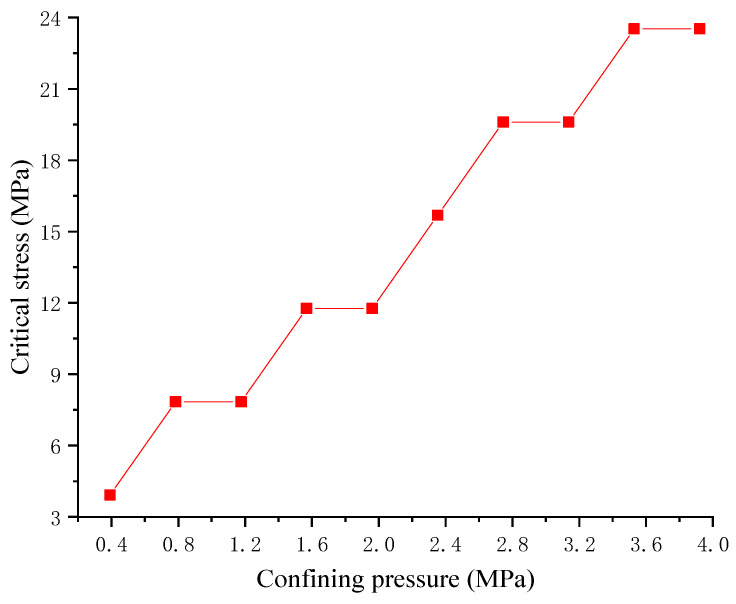
The relationship between confining pressures and critical stress.

For a specific vertical constant load, the creep strain proportional coefficient with confining pressure is smaller than that without confining pressure, and the creep strain proportional coefficient decreases with increasing confining pressure, indicating that confining pressures can effectively reduce creep strain. When the vertical constant load is small, the confining pressure increases the horizontal strain of RAC, resulting in a negative creep strain proportional coefficient. In comparison to the situation without confining pressure, the creep strain proportional coefficient increases with the increase in vertical constant load when confining pressure exists. Therefore, the vertical constant load when the creep strain proportional coefficient changes from negative to positive is taken as the critical stress under this confining pressure. Figure 13 reflects the relationship between the confining pressures and critical stress. The critical stress increases as the confining pressures increase. Therefore, in practical engineering, the critical stress of RAC can be increased by increasing the confining pressures, so as to ensure the long-term stability of RAC.

## 5. Discussion

**Figure 14:**
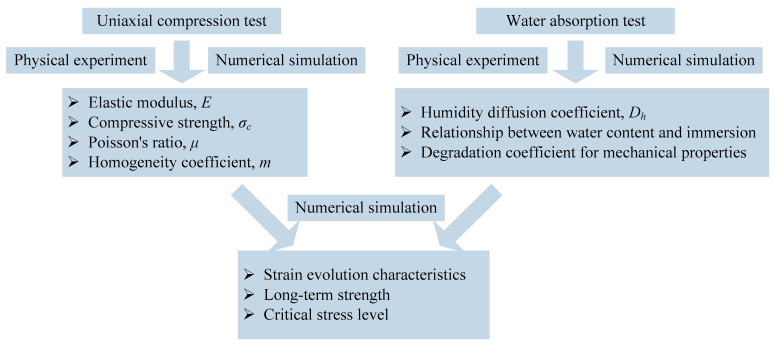
Technical roadmap of this study.

In order to provide a comprehensive study of published papers all over the world regarding the creep of concrete made with recycled aggregates, Lye et al. (2016) [51] carried out a literature search and tried to analyze, evaluate, and synthesize 9200 data matrixes from 27 countries since 1984. Lye et al. (2016) [51] pointed out that the creep of concrete increases at a decreasing rate as the coarse RA increases, and for the 100% coarse RA content, the average increase is 32%. The influencing factors of the creep of concrete made with recycled aggregates include aggregate size and type, the application of pozzolanic cement, the water/cement ratio, design bearing capacity, curing, and loading time. However, the impact of the loading environment has not attracted their attention. Water is one of the most notable factors jeopardizing the durability of RAC [52]. On the one hand, water is a main carrier of aggressive substances [53]. On the other hand, water may lead to particle lubrication and softening, which in turn reduces the mechanical parameters, such as the compressive strength and elastic modulus of RAC [54,55,56,57]. At present, experimental testing is the main method of revealing the influence of water on RAC, which is costly and time-consuming. This study presented numerical technology that can be used to study the long-term strength and deformation of RAC in a water environment. An HSD model presented in RFPA2D-Humidity was used in this study. Firstly, the elastic modulus, compressive strength, Poisson’s ratio, and homogeneity index of the experimental RAC were determined by a uniaxial compression test and a numerical simulation uniaxial compression test. Then, the humidity diffusion coefficient was determined and the correlation between water content and immersion time was studied by water absorption tests and numerical simulation water absorption tests. Secondly, uniaxial compression tests were conducted on samples with various water contents to investigate the change law of elastic modulus and compressive strength with water content, and then to determine the degradation coefficient of material mechanical parameters with water content. The experimental results show that the elastic modulus and compressive strength decrease with increasing water content. Finally, the parameters obtained from the above experiments are invoked in an HSD numerical model to study the time-dependent deformation of RAC in a water environment, and the long-term strength and critical stress for accelerated creep of RAC in a water environment were determined. The research process can be represented by a flow chart in Figure 14.

## 6. Conclusions

Based on the above research method, the main conclusions can be drawn as follows:

(1) The water absorption process can be divided into three distinct stages, i.e., the quick absorption stage, the slow absorption stage, and the stable absorption stage. Furthermore, the water content increased quickly in the first stage and lasted for around 15 h. Then, the rate of water absorption gradually decreased and eventually remained constant after immersion in water for 365 h. In addition, the average water content of the saturated sample was 4.88%.
(2) The elastic modulus and compressive strength decrease as the water content increases. Moreover, when the water content exceeds 4.0%, the elastic modulus and compressive strength decrease significantly.
(3) If the stress level is lower than 60% of the compressive strength of RAC, only the primary creep stage and the secondary creep stage exist, while the tertiary creep stage does not appear. In the primary creep stage, the strain first increases at a decreasing rate then increases with a steady rate, and finally increases with a decreasing rate. The strain–time curve is in the form of a saddle, and the saddle-shaped trend becomes more obvious as the stress level increases. Simultaneously, the strain variation law of the saddle-shaped trend is mainly affected by the evolution law of pores inside the specimen.
(4) If the stress level is higher than 70% of the compressive strength of RAC, the strain increases abruptly with time and the secondary creep stage is not obvious. The model fails quickly if the stress level is higher than 70% of the compressive strength of RAC. The RAC selected in this paper has a long-term strength between 60% and 70% of its compressive strength. Based on the consideration of practical project safety applications, the RAC long-term strength in a water environment is selected as a lower limit with 60% of its compressive strength. In practical engineering, when the stress level is higher than the long-term strength value, the recycled concrete may appear to be in the tertiary creep stage, leading to the failure of the sample. This phenomenon should be paid attention to.
(5) In the absence of confining pressures, the creep strain proportional coefficient decreases first and then increases with the increases in stress level. If the stress level is higher than 20% of the compressive strength of RAC, the proportion of creep strain in the total strain increases, and the creep deformation becomes more and more obvious. Therefore, if the stress level is higher than 20% of the compressive strength of RAC, more attention should be paid to deformation monitoring in practical projects.
(6) Confining pressures can effectively increase the long-term strength and critical stress of the RAC. Furthermore, confining pressures can reduce creep deformation of the RAC. In practical engineering, the long-term stable application of RAC in a water environment can be ensured by increasing the confining pressures.

## Figures and Tables

**Table 1:** Mixed design component content for 1 m^3^ recycled concrete.

Material Usage (kg)						
Water	Cement	Recycled Fine Aggregate	Natural Coarse Aggregate	Mineral Powder	Flyash	Polycarboxylate Superplasticizer
175	240	895	940	60	60	5.8

**Table 2:** Test results of UCTs for experimental recycled concrete.

Sample Number	Elastic Modulus (MPa)	Compressive Strength (MPa)	Poisson’s Ratio
1	12,378.8	38.85	0.24
2	11,949.3	40.04	0.25
3	12,606.1	38.74	0.27
Mean	12,311.4	39.21	0.25

**Table 3:** Numerical simulation results of UCTs with different homogeneity indices.

Homogeneity Index	Elastic Modulus (MPa)	Compressive Strength (MPa)
1	12,088	291.00
2	13,025	50.10
3	12,998	42.60
4	12,868	40.79
5	12,705	39.94
6	12,540	39.45
7	12,383	39.16
8	12,239	38.90

**Table 4:** Parameters used in the water absorption tests of a specimen.

Parameter Name	Value
Homogeneity index	7
Elastic modulus (MPa)	12,311.4
Poisson’s ratio	0.25
Compressive strength (MPa)	39.21
Critical saturation	82%
Initial saturation	55%
Degradation coefficient of compressive strength	0.09
Degradation coefficient of elastic modulus	0.04

**Table 5:** Creep results of the specimen under different stress levels.

Stress Level (MPa)	Initial Strain (10^−3^)	Creep Strain (10^−3^)	Total Strain (10^−3^)
1.9605	0.1594	0.0065	0.1659
3.921	0.3188	0.0129	0.3317
7.842	0.6379	0.0258	0.6637
11.763	0.9566	0.0388	0.9954
15.684	1.2745	0.0519	1.3263
19.605	1.5936	0.0657	1.6593
23.526	1.9199	0.0828	2.0027

## Data Availability

The raw data supporting the conclusions of this article will be made available by the authors on request.
